# Bacterial colonization of central venous catheters after heart valve surgery: a risk factor study

**DOI:** 10.1186/2047-2994-4-S1-P209

**Published:** 2015-06-16

**Authors:** A De La Chapelle, A Mihoubi, N Balarac, M Raucoules-Aime, P Dellamonica

**Affiliations:** 1 Anesthesiology, institut Arnault Tzanck, Saint Laurent du Var, France; 2 Cardiology, institut Arnault Tzanck, France; 3 Biostatistic, institut Arnault Tzanck, France; 4 Anesthesiology, CHU, Nice, France; 5 Infectious diseases, CHU, Nice, France

## Introduction

In the presence of a prosthetic heart valve, the colonization of a central venous catheter (CVC) has been implicated as a risk factor for endocarditis. Avoiding bacterial colonization of Central Venous Catheters (CVCs) is an everyday challenge for clinicians

## Objectives

This study evaluated EuroSCORE (Score use for predicting surgical mortality) and the preoperative presence of diabetes to identify patients at a higher risk of a bacterial contamination of the central venous catheter (CVC) after heart valve replacement or valvuloplasty.

## Methods

An observational evaluation was conducted from January 2006 through January 2013 on prospective data submitted to a database. 1324 consecutive patients after valve surgery were included (from January 2006 to December 2010). The systematic cultures of CVC were performed for all patients regardless of infection symptoms. A long-term monitoring (2 years) was done by phone call (up to January 2013). Patients suspected of prosthetic valve endocarditis had an echocardiography, and a blood analysis (particularly, blood cultures). Statistical analysis was processed by Systat 11 using Chi 2 test; Student’s t test; Kruskal-Wallis test; Fisher exact test and a ‘step-by-step’ logistic regression. A p value < 0.05 was considered statistically significant.

## Results

The catheter-related bacteraemia was 5‰ or 0.84/1000 catheter days. The values of the additive and logistic EuroSCOREs were not significantly higher in case of CVC colonization. EuroSCOREs higher than 6 were significantly but only moderately involved with the occurrence of CVC colonization (p = 0.034) (Odds Ratio 1.76; 95% CI [1.04; 2.97]). Diabetes was significantly but moderately associed with CVC colonization (p = 0.041) (Odds Ratio 1.87; 95% CI [1.02; 3.43]). Among parameters of euroSCORE, an ejection fraction of < 30% was closely related to CVC colonization (p = 0.004) (Odds Ratio 4.91; 95% CI [1.65; 14.55]). The catheter’s exposure influenced significantly catheter colonization.

## Conclusion

A poor left ventricular function is the main risk factor in bacterial colonization of CVC. Global risk score and prexisting diabete are not useful to predict postoperative CVC colonization

## Disclosure of interest

None declared.

